# Monthly Summary

**Published:** 1898-07

**Authors:** 


### Monthly Summary.

COHESIVE AND NON-COHESIVE GOLD.—Nearly every essayist, and dental manufacturer, in designating different kinds of gold, call it "soft or cohesive." I believe one of those terms should be left out of our literature, and that the terms should be definitely "cohesive and non-cohesive." I am sorry that this term "soft gold" sticks to the literature of the profession. It is not a definite term; it does not convey what the essayist wishes to convey. The dental manufacturers have almost forced us to continue these terms by placing them on their product. We may have a gold which is to all practical purposes soft, and still it may be cohesive. Cohesive gold is softer than non-cohesive. You take a piece of plate gold and anneal it, make it soft; the same with a piece of foil gold; you anneal it, and that foil is softer than it was before. The reason that the terms soft and cohesive were used is because a pellet of annealed gold is more "sticky" under the plunger and the mass is not so easily manipulated by pressure as the non-cohesive. But the term soft is a wrong one, and I am in hopes the profession will discard it in the new nomenclature. We have been indefinite in our phraseology and have not conveyed the meaning we wish to convey on account of this indefinitness.

As to the use of non-cohesive gold along the cervical margin, as advocated by many members of the profession, it is used in such a place, not so much because we cannot get adaptation with cohesive gold, but because in placing a layer of non-cohesive gold and condensing the cohesive gold on it, we have a cushion for the cohesive gold to be condensed on, and can mallet over that part of the filling with less danger of injuring the enamel margin than we could if we started with cohesive gold and began our condensation in the early part of the operation. That small piece of non-cohesive gold serves as a cushion to protect the margin from injury from the malleting force. We do not want to use much non-cohesive gold in building up proximal fillings in molars and bicuspids. The only non-cohesive gold I use is the first layer. From that point up I use cohesive gold on account of its greater density. Prof. C. N. Johnson, Chicago, in Illinois Society.—*Dental Brief.*

---

DEATH FROM HODGKINS' DISEASE.—Hodgkins' disease, which caused the death of a Yale student, is a curious, but, fortunately, a comparatively rare affection. It is characterized by the appearance of glandular tumors, first appearing in the neck and armpits and extending in groups throughout other portions of the body. Young adults are the most frequent subjects. The malady is always associated with impoverishment of the blood and the relative increase of its white cells, also with marked enlargement of the spleen and changes in the bone marrow, and generally ends fatally within two years after the first appearance of symptoms. The swellings, which are at first isolated, vary from the size of a bean to that of a hen's egg, and finally multiply and coalesce, forming an almost continuous chain of growth, those encircling the neck being often larger in circumference than the head.

The early removal of the primary enlargements is sometimes beneficial and occasionally curative, but, as a rule, the fundamental error of nutrition which is at the bottom of all the trouble, is scarcely possible of correction by internal remedies. The predisposing causes of the disease are not hereditary in character. In a fair proportion of cases the initiatory swelling of the glands is caused by some comparatively trivial ailment, such as an ulcerated tooth, an inflamed throat or a "running" ear. Life is terminated by exhaustion. Sometimes however, death results from suffocation or from starvation in consequence of obstructive growths in the throat.—*N. Y. Herald.*

---

A STUDENT'S DAMAGE SUIT.—George C. Bollan, whose sensational suit against the Atlanta Dental College, and Dean W. T. Crenshaw was recently announced, had filed a second suit against the college. The first proceeding was a $10,oco suit for libel in that the dean was alleged to have written a highly damaging letter to Bollan's mother, in New Orleans. The second suit, which was filed Tuesday, is for $20,000 damages, and is based on Bollan's dismissal from the college. Bollan claims he had spent $3,000 in attending the college and would have been graduated next March but for the action of the faculty last December in expelling him. Owing to his expulsion from the Atlanta Dental College, he alleges, he cannot be received in any other first-class school of dentistry, and his loss, therefore, will be very great. He also claims the charge that he married a woman of bad character and is living with her in an immoral house is false. He alleges that the nature of the charge has done him great injury, as henceforth no man can be proud to claim his friendship and no good woman be willing to recognize him.

---

DR. DONNALLY would like to see the dental and medical profession more closely united, but expediency forbids. The idea which led to the separation of the two professions has been operating for years. The degree of D. D. S. from the better dental colleges of this country is as high a degree as the M. D. from the medical colleges of equivalent standing. It costs as much time, as much brain, as much hard study, and as much concentration of mind and effort to acquire it. More than this, the holder of the D. D. S. is better qualified to practice his specialty than are the specialists in any other branch of the healing art.—*Cosmos*.

---

FOREIGN DOCTORS AND DENTISTS IN ITALY.—Mr. Frederick H. Balfour writes to the "Times" from Florence :

"It is well known that the Italian Government has for some time past contemplated the disqualification as practitioners in Italy of all foreign medical men who have not taken an Italian degree. This unwise policy, however seems hanging in abeyance, the authorities being apparently reluctant to put it in force in view of the great pecuniary benefits resulting from the presence of English and American visitors and residents, many of whom would be deterred from coming to Italy at all if they were unable to command the services of doctors of their own nationality. But with regard to dentists the government is credited with different views, and it is said that prohibitive enactments are very shortly to be adopted in their case. This would surely be a very short-sighted and unjust step. Italian doctors stand at the head of their profession in Europe, but Italian dentists are greatly inferior, and in many ways far behind the times. The reason probably is that doctors are supposed to be more important to foreign residents than dentists, but surely there are many English people and Americans who are greatly dependent upon dental surgery, and would feel serious misgivings at the idea of living in a country where in case of urgent necessity and suffering they would only have an ignorant and unskillful operator to resort to."

We fail to see why the distinction should be drawn between the doctors and the dentists. We think, moreover, that, as the Italian doctors are good, and the Italian dentists "greatly inferior," it would be a more logical proceeding to reverse the anticipated policy.—*Western Dental Journal*.

